# Community Detection in Complex Networks via Clique Conductance

**DOI:** 10.1038/s41598-018-23932-z

**Published:** 2018-04-13

**Authors:** Zhenqi Lu, Johan Wahlström, Arye Nehorai

**Affiliations:** 1 Preston M. Green Department of Electrical and Systems Engineering, Washington University in St. Louis, St. Louis, MO USA; 2 Department of Computer Science, University of Oxford, Oxford, United Kingdom

## Abstract

Network science plays a central role in understanding and modeling complex systems in many areas including physics, sociology, biology, computer science, economics, politics, and neuroscience. One of the most important features of networks is community structure, i.e., clustering of nodes that are locally densely interconnected. Communities reveal the hierarchical organization of nodes, and detecting communities is of great importance in the study of complex systems. Most existing community-detection methods consider low-order connection patterns at the level of individual links. But high-order connection patterns, at the level of small subnetworks, are generally not considered. In this paper, we develop a novel community-detection method based on cliques, i.e., local complete subnetworks. The proposed method overcomes the deficiencies of previous similar community-detection methods by considering the mathematical properties of cliques. We apply the proposed method to computer-generated graphs and real-world network datasets. When applied to networks with known community structure, the proposed method detects the structure with high fidelity and sensitivity. When applied to networks with no a priori information regarding community structure, the proposed method yields insightful results revealing the organization of these complex networks. We also show that the proposed method is guaranteed to detect near-optimal clusters in the bipartition case.

## Introduction

Networks are a standard representation of complex interactions among multiple objects, and network analysis has become a crucial part of understanding the features of a variety of complex systems^1–10^. One way to analyze networks is to identify *communities*, mesoscopic structures consisting of groups of nodes that are relatively densely connected to each other but sparsely connected to other dense groups in the network^11^. Communities, also called *clusters* or *modules*, mark groups of nodes which could, for example, share common properties, exchange information frequently, or have similar functions within the network^12^. The existence of communities is evident in many networked systems from a great many areas, including physics, sociology, biology, computer science, engineering, economics, politics, and neuroscience^13–20^.

Community detection is important for many reasons. It allows classification of the functions of nodes in accordance with their structural positions in their communities^21–23^. It reveals the hierarchical organization that exists in many real-world networks^24^. Moreover, it improves the performance and efficiency of processing, analyzing, and storing networked data^25,26^. Communities also have concrete applications. In social networks, communities represent groups of individuals with mutual interests and backgrounds, and imply patterns of real social groupings^15^. In purchase networks, communities represent groups of customers with similar purchase habits, and can help establish efficient recommendation systems^26^. In citation networks, communities represent groups of related papers in one research direction, and identify scholars sharing research interests^27^. In brain networks, communities represent groups of nodes that are intricately interconnected and that could perform local computations, and they give insights into structural units of the brain^28^.

The mathematical synonym of networks is *graphs*, and in the context of graph theory, one of the mathematical formalizations of community detection is *graph partitioning*. Guided by spectral graph theory^29^, the method of spectral graph partitioning arose by relating network properties to the spectrum of the Laplacian matrix^30^. The earliest method in this category minimized connections between different communities^31,32^. In practice, this optimization problem can be efficiently solved, but it favors non-optimal solutions involving cutting a small part from the graph. One way to circumvent this drawback is to introduce balancing factors to the objective functions in order to enforce a reasonably large size for each community^33,34^. However, introducing balancing factors makes these optimization problems NP-hard^35^. Hence relaxed versions of these problems are solved by taking advantage of the properties of the Laplacian matrix.

Despite thousands of publications in the literature on spectral partitioning, these methods are constrained to conventional graph-based models. These models involve a set of vertices, which represent objects of interest, and a set of edges, which encode the existence or non-existence of a relationship between each pair of objects. However, in many real-world systems, the complex and rich nature of systems cannot be captured by such *dyadic* relationships. More importantly, recent computer innovations have greatly increased the size of the real networks that one can potentially handle. As a result, the way to process and understand graphs has been changed, and *polyadic* interactions are becoming more and more important. In particular, a community is intuitively a cohesive group of vertices that are “more densely” connected within the community than across communities^11^. The precise definition and characterization of “more densely” relies on polyadic interactions among multiple vertices. In order to quantitatively characterize polyadic structures, we employ the high-order structures of *cliques*, defined to be local complete subgraphs. In the context of networks, cliques are groups of objects that rapidly and effectively interact. This paper presents a graph-partitioning method that identifies clusters of cliques.

One line of related work is the method of *k*-clique percolation^36,37^. This method defines the *k*-clique community to be the union of “adjacent” *k*-cliques, which by definition share *k* − 1 vertices, where *k* is any positive integer. However, this definition is too stringent because it rules out other possible communities that are not so well-connected. Its performance also relies heavily on the choice of *k*: A small *k* leads to a single giant community, and a large *k* leads to multiple small and possibly distant communities. In addition, this definition includes topological cavities^38^, which enclose holes in networks and mark local lacks of connectivity. However, this feature is not an expected property of communities.

In a recent paper, Benson *et al*. devised a community-detection method based on high-order connectivity patterns called network motifs^39,40^, and proposed a generalized framework for identifying clusters of network motifs^41^. Cliques are certainly one special kind of network motif, and Benson *et al*. provide numerical simulations for applying this framework to cliques. However, this framework has several drawbacks. First, the framework fails to consider the nested nature of cliques and so suffers from unnecessary computational cost, since it needs to take into consideration non-maximal cliques. Second, the method requires pre-specification of the sizes of the cliques involved, instead of considering all clique sizes occurring in the network. Third, the conductance function merely counts the number of cliques and ignores other properties influenced by partitions. Lastly, the performance guarantee works only for 3-cliques. We overcome all these drawbacks by designing a novel conductance function specifically for cliques.

In this paper, we propose a novel community-detection method that minimizes a new objective function, called the clique conductance function. We encode in this objective function the number and sizes of cliques, and the numbers of edges in the cliques. Finding a partition that exactly minimizes the clique conductance is computationally intractable. Thus we extend the spectral graph partitioning methodology, and devise a computationally tractable solution that approximately minimizes the clique conductance. In addition, we derive a performance guarantee for the bipartition case, showing that the resulting bipartition is near-optimal. Finally, we apply the proposed method to computer-generated graphs and real-world network datasets. When applied to networks with known community structure, the proposed method achieves excellent agreement with the ground-truth communities. When applied to networks with no a priori information regarding community structure, the proposed method yields insightful results that help us understand the structures embedded in these complex networks.

## Methods

In this section, we describe our proposed graph-partitioning method. We begin by introducing several graph notations, and then state the formulation of our proposed graph-partitioning method based on clique-conductance minimization. We conclude this section by proposing a computationally efficient algorithm that approximately solves the optimization problem.

### Graph Notations

An undirected weighted graph ${\mathcal{G}}$ is an ordered triplet $({\mathcal{V}},{\mathcal{E}},\pi )$ consisting of a set of vertices ${\mathcal{V}}=\{{v}_{1},\ldots ,{v}_{n}\}$, a set of edges ${\mathcal{E}}\subset {\mathcal{V}}\times {\mathcal{V}}$ satisfying $(u,v)\in  {\mathcal E}$ if and only if $(v,u)\in  {\mathcal E}$ for all $u,v\in {\mathcal{V}}$, and a weight function $\pi :{\mathcal{V}}\times {\mathcal{V}}\to {{\mathbb{R}}}^{+}\cup \{0\}$ satisfying $\pi ( {\mathcal E} )\, > \,0$, $\pi ({\mathcal{V}}\times {\mathcal{V}}-{\mathcal{E}})\,=\,0$, and *π*(*u*, *v*) = *π*(*v*, *u*) for all $u,v\in {\mathcal{V}}$. If the weight function *π* in addition satisfies $\pi ( {\mathcal E} )=1$, then ${\mathcal{G}}$ is an undirected binary graph. The weighted adjacency matrix **W** of the graph is defined as **W**(*i*, *j*) := *π*(*v*_*i*_, *v*_*j*_). Since ${\mathcal{G}}$ is undirected, we have **W** = **W**^T^. The degree of a vertex *v*_*i*_ is defined as ${d}_{i}\,:={\sum }_{u\in {\mathcal{V}}}\pi (u,{v}_{i})$, and the degree matrix **D** is a diagonal matrix with *d*_1_, …, *d*_*n*_ as diagonal entries. The Laplacian matrix **L** of the graph is defined as **L** := **D** − **W**. A graph ${\mathcal{G}}$ is said to have no loops if *π*(*u*, *u*) = 0 for all $u\in {\mathcal{V}}$.

Formally, a *k*-clique is a subgraph consisting of *k* nodes with all pairwise connections, where *k* is any positive integer. It naturally follows from the definition that any subgraph of a clique is also a clique, and such a subgraph is called a *face*. We call this feature the *nested nature* of cliques. A *maximal* clique is a clique that is not a face. Due to the nested nature of cliques, the maximal cliques of a graph contain all the clique information. The number of vertices constituting a clique *σ* is called the size of a clique and is denoted as *ω*(*σ*). In this paper, we use ${ {\mathcal M} }_{k}$ to represent the collection of all maximal *k*-cliques, and ${\mathcal{M}}={\bigcup }_{k}{{\mathcal{M}}}_{k}$ to represent the collection of all maximal cliques.

### Clique Conductance Minimization

We now state the formulation of our proposed graph-partitioning method. Intuitively, the graph-partitioning problem based on cliques can be described as follows: We wish to find a partition of the graph, such that cliques between different groups are few and have small sizes (which means that vertices in different clusters share few high-order connections), and cliques within each group have large sizes (which means that vertices within one cluster are connected in high-order fashion). Formally, suppose that ${\mathcal{G}}=({\mathcal{V}},\,{\mathcal{E}},\,\pi )$ is an undirected binary graph with no loops. Given a positive integer *m* > 1, we wish to find a partition (*A*_1_, …, *A*_*m*_) that satisfies *A*_*i*_∩*A*_*j*_ = ∅ for any *i* ≠ *j* and ${\bigcup }_{i}{A}_{i}={\mathcal{V}}$, and that minimizes

1 $$\psi ({A}_{1},\,\ldots ,\,{A}_{m})\,:=\,\sum _{i=1}^{m}\,{\rm{c}}{\rm{u}}{\rm{t}}\,({A}_{i},\,{\bar{A}}_{i}),$$

where

2 $${\rm{c}}{\rm{u}}{\rm{t}}\,(A,\bar{A})\,:=\,\sum _{\sigma \in {\mathcal{M}}}\omega (\sigma )\sum _{u,v\in \sigma }{\mathbbm{1}}(u\in A,\,v\in \bar{A}),$$

where ${\mathbbm{1}}$ is the truth-value indicator function. Conceptually, the cut function ${\rm{c}}{\rm{u}}{\rm{t}}(A,\,\bar{A})$ measures how severely maximal cliques are influenced by the partition $(A,\,\bar{A})$. The cut function considers both the number and sizes of maximal cliques that are cut by the partition, and also the number of edges in each maximal clique that are cut by the partition. Unfortunately, in practice the solution of this approach often yields extreme cases separating the vertex with the lowest degree from the rest of the graph, similar to phenomena observed in minimizing conventional cut functions^31^. To circumvent this problem, we introduce a balancing factor

3 $${\rm{vol}}(A)\,:\,=\,\sum _{\sigma \in  {\mathcal M} }\omega (\sigma )\sum _{u\in \sigma }{\mathbbm{1}}(u\in A),$$

which conceptually measures the size of a cluster *A*, and propose to minimize the clique conductance function defined as

4 $$\varphi ({A}_{1},\,\ldots \,,{A}_{m})\,:=\,\sum _{i=1}^{m}\frac{{\rm{c}}{\rm{u}}{\rm{t}}({A}_{i},{\bar{A}}_{i})}{min({\rm{v}}{\rm{o}}{\rm{l}}({A}_{i}),{\rm{v}}{\rm{o}}{\rm{l}}({\bar{A}}_{i}))}.$$

We note that this objective function is formulated in a similar way to normalized spectral partitioning^34^. However, introducing balancing factors causes the computationally tractable problem of minimizing equation (1) to become NP-hard^35^. Following the idea of spectral graph partitioning^30^, we next reformulate our optimization problem and seek a computationally tractable solution.

### Partitioning Algorithm

We introduce a new weighted graph, which we call the induced clique graph, to encode the maximal-clique information of ${\mathcal{G}}$. The induced clique graph of ${\mathcal{G}}=({\mathcal{V}},{\mathcal{E}},\pi )$ is an undirected weighted graph ${{\mathcal{G}}}_{c}=({\mathcal{V}},{\mathcal{E}},{\pi }_{c})$, where the weight function *π*_*c*_ is defined as

5 $${{\pi }}_{c}(u,v)\,:\,=\,\sum _{\sigma \in  {\mathcal M} }\sum _{u,v\in \sigma }\omega (\sigma \mathrm{).}$$

By definition, *π*_*c*_(*u*, *v*) is the sum of the sizes of the maximal cliques that vertex *u* and vertex *v* both engage. Intuitively, *π*_*c*_ measures how densely two vertices are connected in ${\mathcal{G}}$. We denote by **W**_*c*_, **D**_*c*_, **L**_*c*_ the corresponding adjacency matrix, degree matrix, and Laplacian matrix, respectively. Following this spirit, the graph-partitioning problem on an undirected binary graph ${\mathcal{G}}$ can be transformed and implemented as a graph-partitioning problem on a weighted graph ${{\mathcal{G}}}_{c}$. Notice that a partition (*A*_1_, …, *A*_*m*_) on the original network ${\mathcal{G}}$ induces a partition on the induced clique graph ${{\mathcal{G}}}_{c}$. To measure conductance on this weighted graph, we recall the traditional conductance function on weighted graphs^30^, defined as

6 $${\varphi }_{c}({A}_{1},\,\ldots ,\,{A}_{m})\,:=\,\sum _{i=1}^{m}\frac{{{\rm{c}}{\rm{u}}{\rm{t}}}_{c}({A}_{i},{\bar{A}}_{i})}{min({{\rm{v}}{\rm{o}}{\rm{l}}}_{c}({A}_{i}),{{\rm{v}}{\rm{o}}{\rm{l}}}_{c}({\bar{A}}_{i}))},$$

where

7 $${{\rm{c}}{\rm{u}}{\rm{t}}}_{c}(A,\bar{A})\,:=\,\sum _{u\in A,v\in \bar{A}}{\pi }_{c}(u,v)$$

is the total weight of edges cut, and

8 $${{\rm{v}}{\rm{o}}{\rm{l}}}_{c}(A)\,:=\,\sum _{u\in A,v\in {\mathcal{V}}}{\pi }_{c}(u,v)$$

is the total connection from vertices in *A* to all vertices in the graph. The next proposition relates the traditional conductance function in equation (6) to the clique conductance function in equation (4).

**Algorithm 1:**
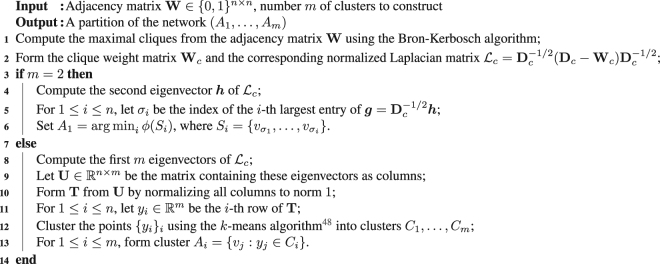
Graph partitioning via clique conductance minimization.

#### Proposition 1.

*Given any undirected binary graph* ${\mathcal{G}}=({\mathcal{V}},{\mathcal{E}},\pi )$*, for any subset* $A\subset {\mathcal{V}}$, we have

9 $${\rm{c}}{\rm{u}}{\rm{t}}(A,\bar{A})={{\rm{c}}{\rm{u}}{\rm{t}}}_{c}(A,\bar{A}),$$

10 $${\rm{vol}}(A)={{\rm{vol}}}_{c}(A).$$

The proof of Proposition 1 is given later. A straightforward consequence of Proposition 1 is that the conductance functions as shown in equations (4) and (6) are equal.

#### Corollary 2.

*Given any undirected binary graph* ${\mathcal{G}}=({\mathcal{V}},{\mathcal{E}},\pi )$*, for any natural number m* > 1 *and any partition (A*_1_, …, *A*_*m*_*), we have*

11 $${\varphi }({A}_{1},\ldots ,{A}_{m})={{\varphi }}_{c}({A}_{1},\ldots ,{A}_{m}\mathrm{).}$$

Corollary 2 shows that the clique conductance minimization problem,

12 $$\mathop{{\rm{minimize}}}\limits_{({A}_{1},\ldots ,{A}_{m})}\,{\varphi }({A}_{1},\ldots ,{A}_{m}),$$

is equivalent to the conductance minimization problem on the induced weighted graph,

13 $$\mathop{{\rm{minimize}}}\limits_{({A}_{1},\ldots ,{A}_{m})}\,{{\varphi }}_{c}({A}_{1},\ldots ,{A}_{m}\mathrm{).}$$

Solving this minimization problem directly can be computationally intractable^35^. One way to circumvent this issue is to solve a relaxed version of this problem by employing normalized spectral partitioning^30,34,42^. Thus our partitioning algorithm consists of three steps. First the maximal cliques are computed using the Bron-Kerbosch algorithm^43–46^. Then the induced clique graph ${{\mathcal{G}}}_{c}$ is formed. Finally, normalized spectral partitioning^42^ is applied to achieve a partition of the graph ${\mathcal{G}}$. Our partitioning algorithm is stated in detail in Algorithm 1. As shown in Algorithm 1, we use two different clustering methods for *m* = 2 and *m* > 2 when applying normalized spectral partitioning, because for *m* = 2 the Cheeger inequality ensures that this clustering method produces a near-optimal partition, as shown later. For the general case of *m* > 2, there are no similar results providing performance guarantees. Among the several spectral partitioning methods^30^, we choose normalized spectral partitioning^42^ because of the construction of the clique conductance function. A recent work provides a performance guarantee for the general case, but the proof is constrained to regular binary graphs and is based on a new and untested clustering method^47^. We choose to keep using the *k*-means clustering method for its ease of implementation and successful empirical results.

## Empirical Results

In this section we present a number of numerical experiments with the proposed method. We first perform experiments on computer-generated graphs, and then apply the proposed method to real-world networks with known community structures. In each case, we find that the proposed method almost perfectly detects community structures indicated by network connectivity.

### Benchmarks

We use benchmarks to compare the proposed method to the motif-conductance method^41^, the normalized spectral partitioning^34^, and greedy methods, including the Louvain method^48^, the Ravasz method^49^, and the fast modularity maximization method^50–52^. Benchmarks are computer-generated graphs whose community structure is known. To compare two partitions ${{\mathcal{C}}}_{1},{{\mathcal{C}}}_{2}$ of the same graph, we use the normalized mutual information^53,54^, defined as

14 $${I}_{n}({{\mathcal{C}}}_{1},{{\mathcal{C}}}_{2})\,:=\,\frac{{\sum }_{{c}_{1}\in {{\mathcal{C}}}_{1},{c}_{2}\in {{\mathcal{C}}}_{2}}p({c}_{1},{c}_{2}){\rm{l}}{\rm{o}}{\rm{g}}\,\frac{p({c}_{1},{c}_{2})}{p({c}_{1})p({c}_{2})}}{\frac{1}{2}{\mathcal{H}}({{\mathcal{C}}}_{1})+\frac{1}{2}{\mathcal{H}}({{\mathcal{C}}}_{2})}.$$

Here, *p*(*c*) is the probability that a randomly chosen vertex belongs to community *c*, *p*(*c*_1_, *c*_2_) is the probability that a randomly chosen vertex belongs to both community *c*_1_ and community *c*_2_. Also, ${\mathcal{H}}({\mathcal{C}})$ is the Shannon entropy, defined as

15 $${\mathcal{H}}({\mathcal{C}})\,:=\,-\sum _{c\in {\mathcal{C}}}p(c)\,{\rm{l}}{\rm{o}}{\rm{g}}\,p(c).$$

Intuitively, the normalized mutual information measures the similarity between two partitions. If the two partitions ${{\mathcal{C}}}_{1},\,{{\mathcal{C}}}_{2}$ are identical, then ${I}_{n}({{\mathcal{C}}}_{1},\,{{\mathcal{C}}}_{2})=1$, and if the two partitions are independent of each other, then ${I}_{n}({{\mathcal{C}}}_{1},\,{{\mathcal{C}}}_{2})=0$. In the following experiments, ${{\mathcal{C}}}_{1}$ is the ground-truth partition given by the benchmark, and ${{\mathcal{C}}}_{2}$ is the partition predicted by a community-detection method.

The first benchmark we use is the Girvan-Newman (GN) benchmark^55^. Here, each graph is composed of 128 vertices and is partitioned into 4 communities of size 32. Each vertex is connected to approximately 16 others. For each vertex, a fraction *z*_out_ of 16 connections is made to randomly chosen vertices of other communities, and the remaining connections are made to randomly chosen members of the same community. When *z*_out_ is a half-integer $k+\frac{1}{2}$, half of the vertices have *k* inter-community connections and the other half have *k* + 1 inter-community connections. The GN benchmark produces graphs with known community structures, which are essentially random in all other aspects.

**Figure 1:**
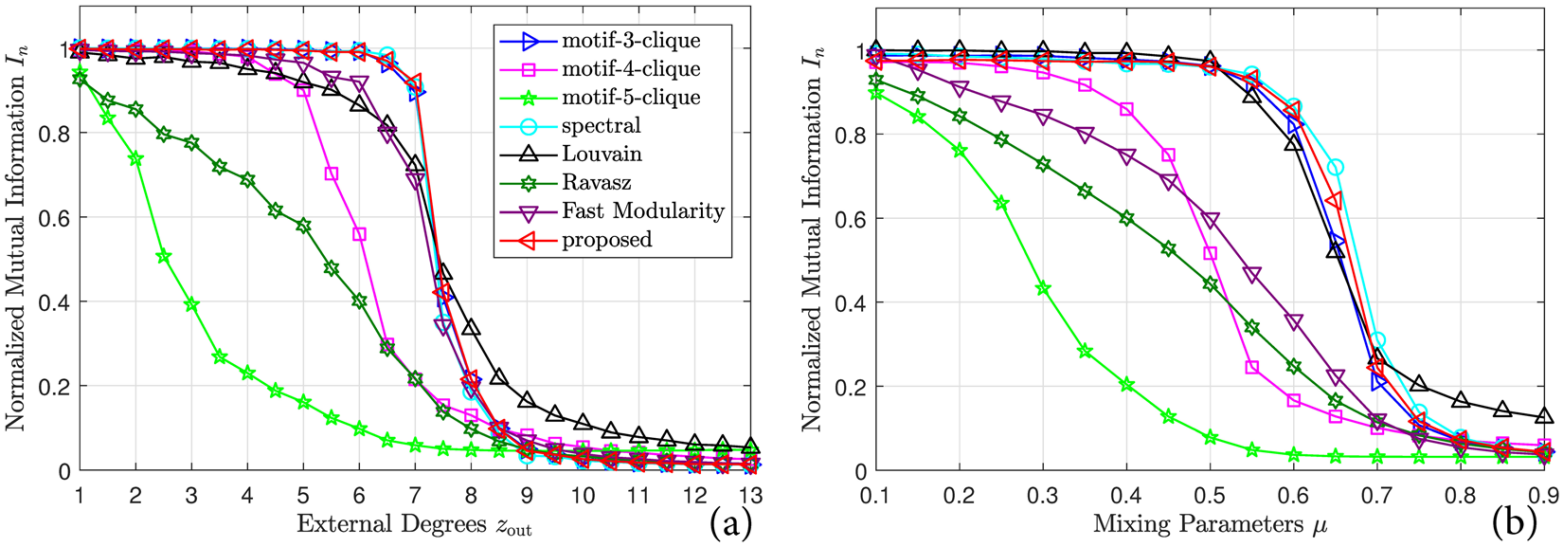
Normalized mutual information of different community-detection methods on (**a**) the Girvan-Newman benchmark, (**b**) the Lancichinetti-Fortunato-Radicchi benchmark (using the same legend as subfigure (a)).

The results of different community-detection methods compared against the GN benchmark are shown in Fig. 1a. Each curve is averaged over 1000 realizations. As can be seen, the proposed method achieves complete mutual information when *z*_out_ ≤ 7, detecting virtually correct communities. The proposed method yields almost zero mutual information when *z*_out_ ≥ 9, where each vertex has more inter-community connections than intra-community connections. The transition between these two regions is swift and sharp. In other words, the proposed method performs almost perfectly up to the point where each vertex has as many inter-community connections as intra-community connections. This performance is almost optimal, because the ground-truth community structure diminishes when each vertex has more inter-community connections than intra-community connections. In this situation, the community structure represented by graph connections deviates from the ground-truth community structure, and so these two sets of clusters share little mutual information. The normalized spectral partitioning and the motif-conductance method using 3-cliques as the network motif perform as well as the proposed method. But when 4-cliques and 5-cliques are chosen as network motifs, the performance of the motif-conductance method degrades severely. This degradation shows that the motif-conductance method heavily relies on the choice of, and prior knowledge about, which cliques are overexpressed in a graph. Finding this knowledge and determining this choice necessarily involve a brute-force search over all subgraphs of certain sizes. Among the greedy methods, the Louvain method and the fast modularity method offer the best performance, but compared to the proposed method, the accuracies of both methods are lower when *z*_out_ ≤ 7.

The GN benchmark generates a random graph where all vertices have approximately same degrees and all communities have an identical size. However, many real-world networks are scale-free^56^, with node degrees and community sizes following the power-law distribution. As a result, a community-detection method that performs well on the GN benchmark might fail on real-world networks. To ensure that the proposed method does not suffer from this limitation, we use the Lancichinetti-Fortunato-Radicchi (LFR) benchmark as a second benchmark^57^, where both vertex degrees and ground-truth community sizes follow the power-law distribution. In this benchmark, each graph is composed of *n* vertices and is partitioned into *m* communities. Each vertex is given a degree following a power-law distribution with exponent *γ*, and each community is given a size following a power-law distribution with exponent *β*. The minimal and maximal values of degrees, *k*_min_, *k*_max_, and of community sizes, *s*_min_, *s*_max_, are chosen such that *k*_min_ < *s*_min_ and *k*_max_ < *s*_max_. For each vertex, a fraction 1 − *μ* of its connections is made to randomly chosen members of the same community, and the remaining connections are made to randomly chosen members of other communities. A realization of this benchmark is constructed via the following steps. At the beginning, all vertices are homeless, i.e., they belong to no communities. Each vertex is assigned to a randomly chosen community with a size greater than the vertex degree. If the community is already full, a randomly chosen member of this community is kicked out. This procedure continues until each vertex is assigned to a community. Then connections are randomly generated while preserving the ratio between the external and internal degrees of each vertex.

The results of different community-detection methods compared against the LFR benchmark are shown in Fig. 1b, with parameters chosen as *n* = 500, *m* = 10, *k*_min_ = 20, *k*_max_ = 80, *γ* = 2, *s*_min_ = 30, *s*_max_ = 100, and *β* = 1.1. Each curve is averaged over 1000 realizations. The results are similar to those on the GN benchmark. The proposed method, the normalized spectral partitioning method, and the motif-conductance method using 3-cliques perform similarly: All closely approximate complete mutual information when *μ* ≤ 0.5, and yield nearly zero mutual information when *μ* ≥ 0.8. The performance of the motif-conductance method degrades severely when 4-cliques and 5-cliques are chosen as network motifs. The performances of the greedy methods are similar to their performances on the GN benchmark, except that the fast modularity method has a much lower accuracy when *μ* ≤ 0.6.

**Figure 2:**
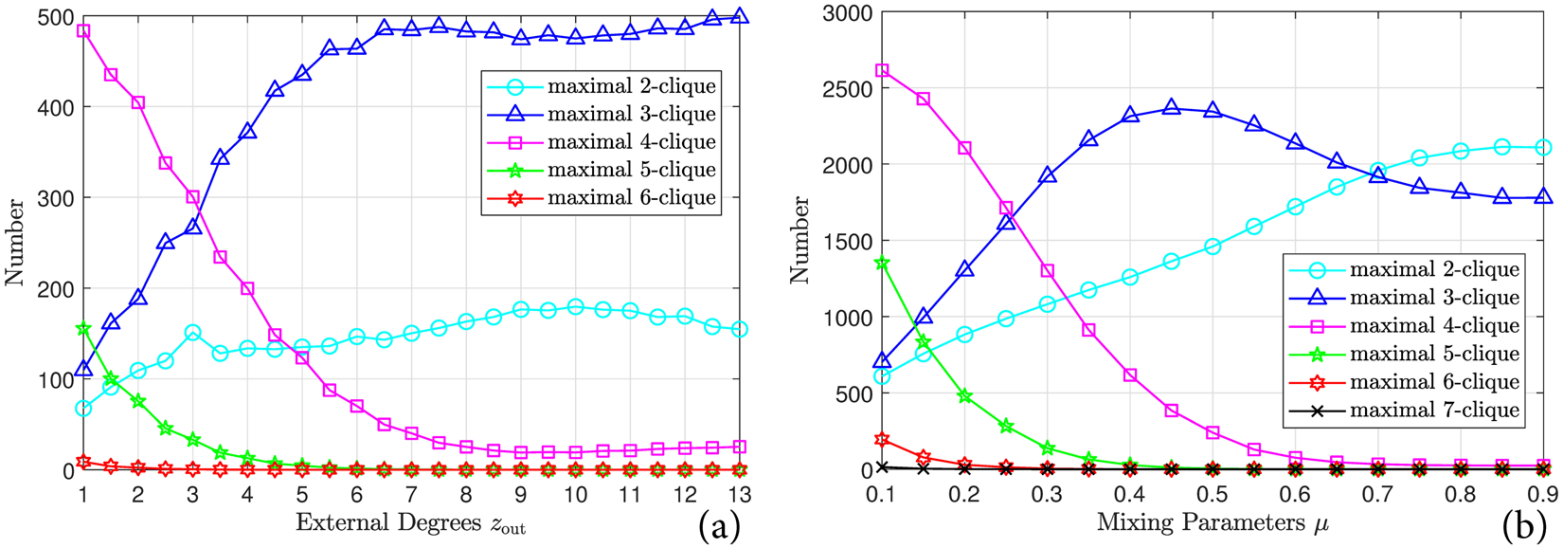
Distribution of sizes of maximal cliques in (**a**) the Girvan-Newman benchmark, (**b**) the Lancichinetti-Fortunato-Radicchi benchmark.

To further validate the advantage of the proposed method over the motif-conductance method, we depict in Fig. 2 the size distribution of maximal cliques in both benchmarks averaged over 1000 realizations. The distributions in both benchmarks are similar. When *z*_out_ and *μ* are small, the 4-cliques are the dominant maximal cliques and other maximal cliques generally have sizes of 2, 3, and 5. With increasing *z*_out_ and *μ*, the numbers of 4-cliques and 5-cliques decrease rapidly and are exceeded by the numbers of 2-cliques and 3-cliques when approximately 1/3 of the connections of each vertex are inter-community. In the GN benchmark, the number of 3-cliques keeps growing after this point and remains the most numerous maximal clique. But in the LFR benchmark, the number of 3-cliques is exceeded by the number of 2-cliques when *μ* > 0.7. Given these patterns in the distributions, it is not surprising that the motif-conductance method performs poorly when 4-cliques and 5-cliques are chosen as network motifs. These distributions also further demonstrate the advantage of the proposed method. In practice, the distribution of cliques (and other network motifs) is mostly probably unavailable when one is processing observed network data. Collecting this information is computationally expensive. Since the maximal cliques contain all the clique information, the proposed method is able to process general networks with no prior knowledge of clique sizes and clique locations.

In summary, the proposed method achieves state-of-the-art performance on the homogeneous GN benchmark and on the scale-free LFR benchmark. In addition, the proposed method yields almost the optimal performance one could expect on these two benchmarks: The proposed method detects the pre-defined ground-truth community structure when it is well represented by connections, and deviates from it when the ground truth diminishes. This behavior explains why there is little improvement over the existing methods. As opposed to the motif-based method, the proposed method also benefits from the fact that it requires no pre-specification of clique sizes. As a result, the proposed method bypasses a computationally expensive search for the optimal choice of clique sizes.

### Zachary’s Karate Club

**Figure 3:**
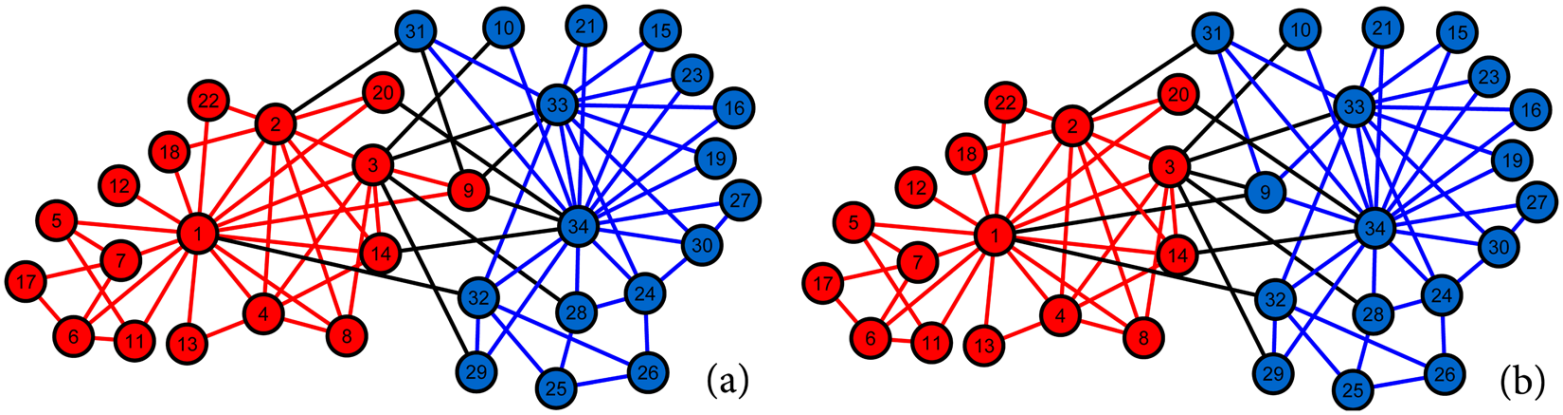
The friendship network from Zachary’s karate club study. (**a**) The communities observed by Zachary. (**b**) The communities detected by the proposed method.

We apply our method to the network from the well-known karate club study by Zachary^58^. This study followed a social network composed of 34 members and 78 pairwise links observed over a period of three years. During the study, a political conflict arose between the club president (node 34) and the instructor (node 1). This political conflict later caused the club to split into two parts, each with half of the members. Zachary recorded a network of friendships among members of the club shortly before the fission, and a simplified unweighted version is shown in Fig. 3a. Different node colors are used in this figure to show the two factions of the fission after the political conflict.

Figure 3b shows the community structure detected by the proposed method. The identified communities almost perfectly reflect the two factions observed by Zachary, with only 1 (node 9) out of 34 nodes “incorrectly” assigned to the opposing faction. This exception can be explained by the conflict of interest faced by individual number 9. As recorded by Zachary, individual number 9 was a weak political supporter of the club president before the fission, but not solidly a member of either faction^58^. This ambivalence is revealed by the fact that node 9 is engaged in two maximal 3-cliques, on nodes {1, 3, 9} and on nodes {3, 9, 33}, and one maximal 4-clique on nodes {9, 31, 33, 34}, implying that node 9 is weakly more densely associated with members of the club president’s faction. On the other hand, Zachary pointed out that individual number 9 had an overwhelming interest in staying associated with the instructor, which was not shared by any other member of the club. Individual number 9 was facing his black-belt exam in three weeks, and joining the club president’s faction would result in renouncing his rank and starting over again^58^. In other words, individual number 9 would have joined the club president’s faction, if this conflict of interest had not emerged. Therefore, the proposed method perfectly detected the social communities in an empirically observed network of friendships.

### College Football Network

**Figure 4:**
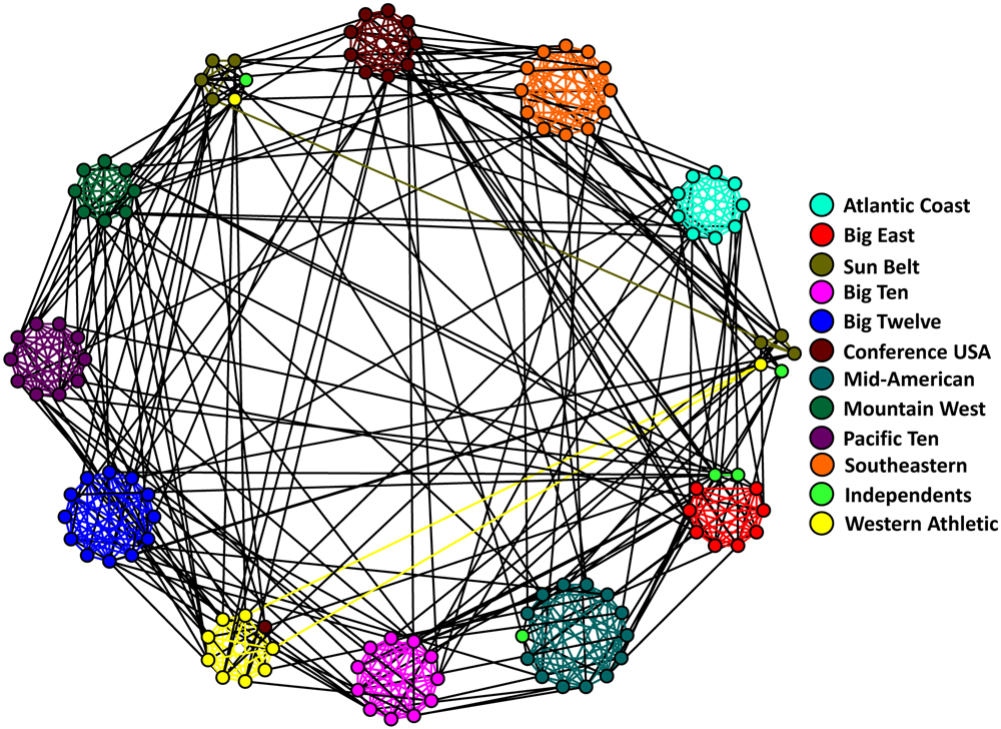
Communities of college football network, using colors for conferences and spatial clusterings for identified communities.

We then apply the proposed method to a more complex real-world network with known community structures. The network represents the schedule of United States football games between Division IA colleges during the regular season in Fall 2000^55^. The network is shown in Fig. 4, where the nodes represent teams, and the links represent regular season games between the two teams connected. The known communities are defined by conferences, each containing around 8 to 12 teams and marked with colors. Links representing intra-conference games are also marked with the same colors as the corresponding conferences. In principle, teams from one conference are more likely to play games with each other than with teams belonging to different conferences. There also exist some independent teams that do not belong to any conference, and these teams are marked with a light-green color.

The communities identified by the proposed method are represented by spatial clusterings in Fig. 4. In general, the proposed method correctly clusters teams from one conference. The independent teams are clustered with conferences with which they played games most frequently, because the independent teams seldom play games between themselves. The clusters detected by the proposed method deviate from the conference segmentation in several ways. First, the Sun Belt conference, marked with a brown color, is split into two parts, shown at the eleven o’clock and three o’clock directions, and each part is grouped with teams from the Western Athletic conference, marked with a yellow color, and independent teams. But this result is understandable given the fact that there was only one game involving teams from both these two parts. Second, one team from the Conference USA conference, marked with a dark red color, is clustered with teams from the Western Athletic conference. This team played no games with other teams from the Conference USA conference, but played games with every team from the Western Athletic conference. Third, two teams from the Western Athletic conference are isolated from other teams from this conference, and each is grouped with part of the Sun Belt conference. The team at eleven o’clock had no intra-conference game, and the team at three o’clock had only two intra-conference games, but they had inter-conference games with every member of the cluster that they are assigned to. In summary, the proposed method perfectly reflected the community structures established in regular-season-game association, and in addition detected the lack of intra-conference association that the known community structure fails to represent.

## Applications to Complex Real-World Networks

In the previous section, we tested the proposed method on both computer-generated graphs and real-world networks for which the community structures are well-defined and known a priori. In this section, we apply the proposed method to complex real-world networks of which the community structures are not known, and show that the proposed method helps us understand these complex networks. For each application example, the number of communities is chosen based on prior information regarding the datasets.

### Bottlenose Dolphin Social Network

**Figure 5:**
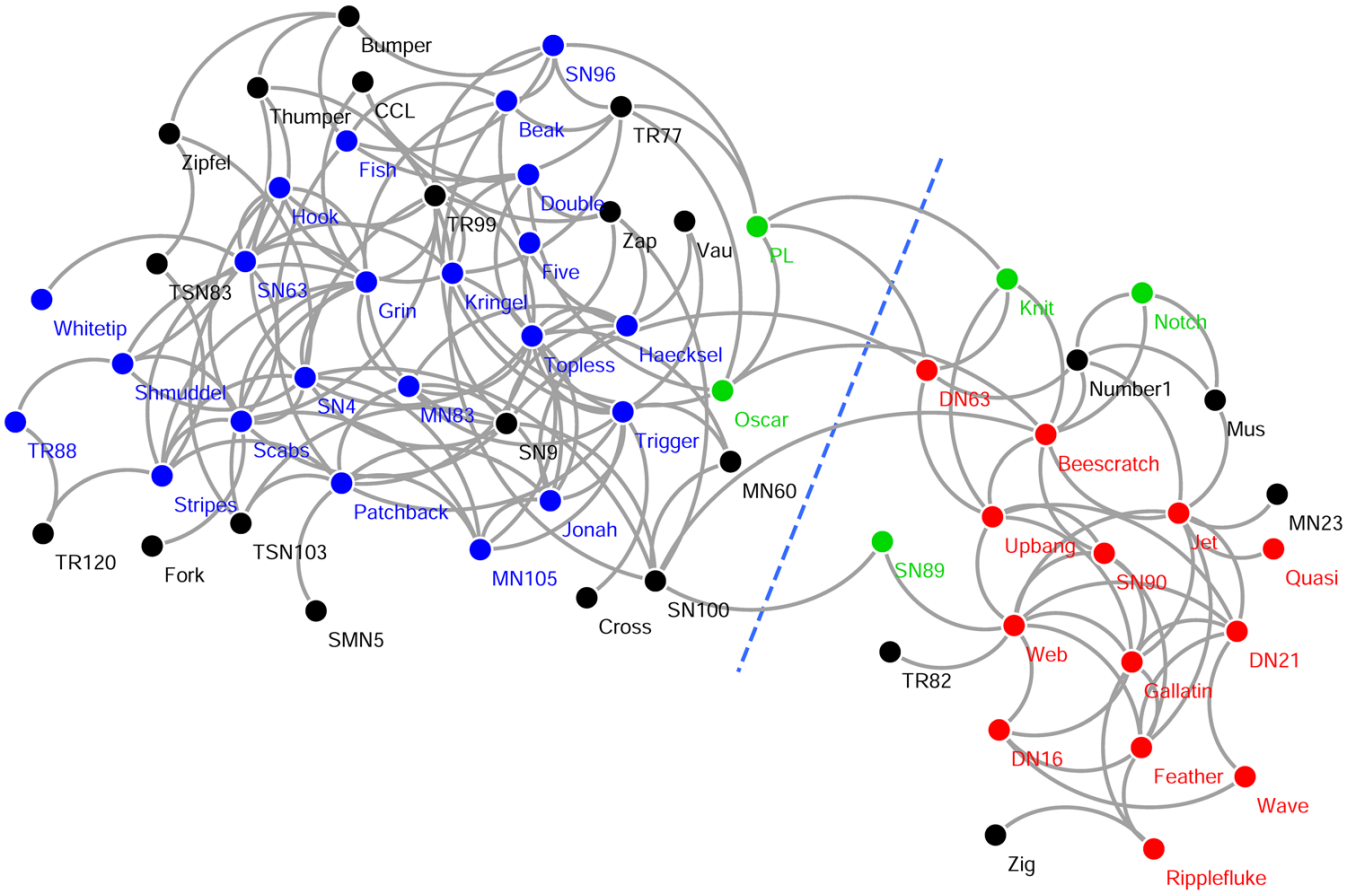
Social network of 62 bottlenose dolphins. The nodes are colored based on the groups observed in the study by Lusseau *et al*.^59^. The spatial clustering represents communities detected by the proposed method.

Our first example is a social network composed of 62 bottlenose dolphins living in Doubtful Sound, New Zealand^59^. The social ties between dolphin pairs are established based on direct observations conducted during a period of seven years by Lusseau *et al*. The clustering analysis conducted by Lusseau *et al*. on 40 of these dolphins shows that three groups spent more time together than all individuals did on average, but group 1 is relatively weak in the sense that it is an artifact of the similar likelihood of encountering these individuals in the study area^59^. Figure 5 shows the social network of bottlenose dolphins, where nodes represent dolphins and links represent social ties. The three groups observed by Lusseau *et al*. are colored in green, red, and blue, respectively, and the dolphins not involved in the clustering analysis by Lusseau *et al*. are left in black. The dashed line denotes the community division found by the proposed method. As can be seen, the achieved division corresponds well with the observed groups, separating the red and blue groups into two communities. The green group (group 1) is split evenly between the two detected communities. This phenomenon is understandable, because group 1 is a weak group and is not well represented by the social network since most of its members share no social ties.

### Food Web

**Figure 6:**
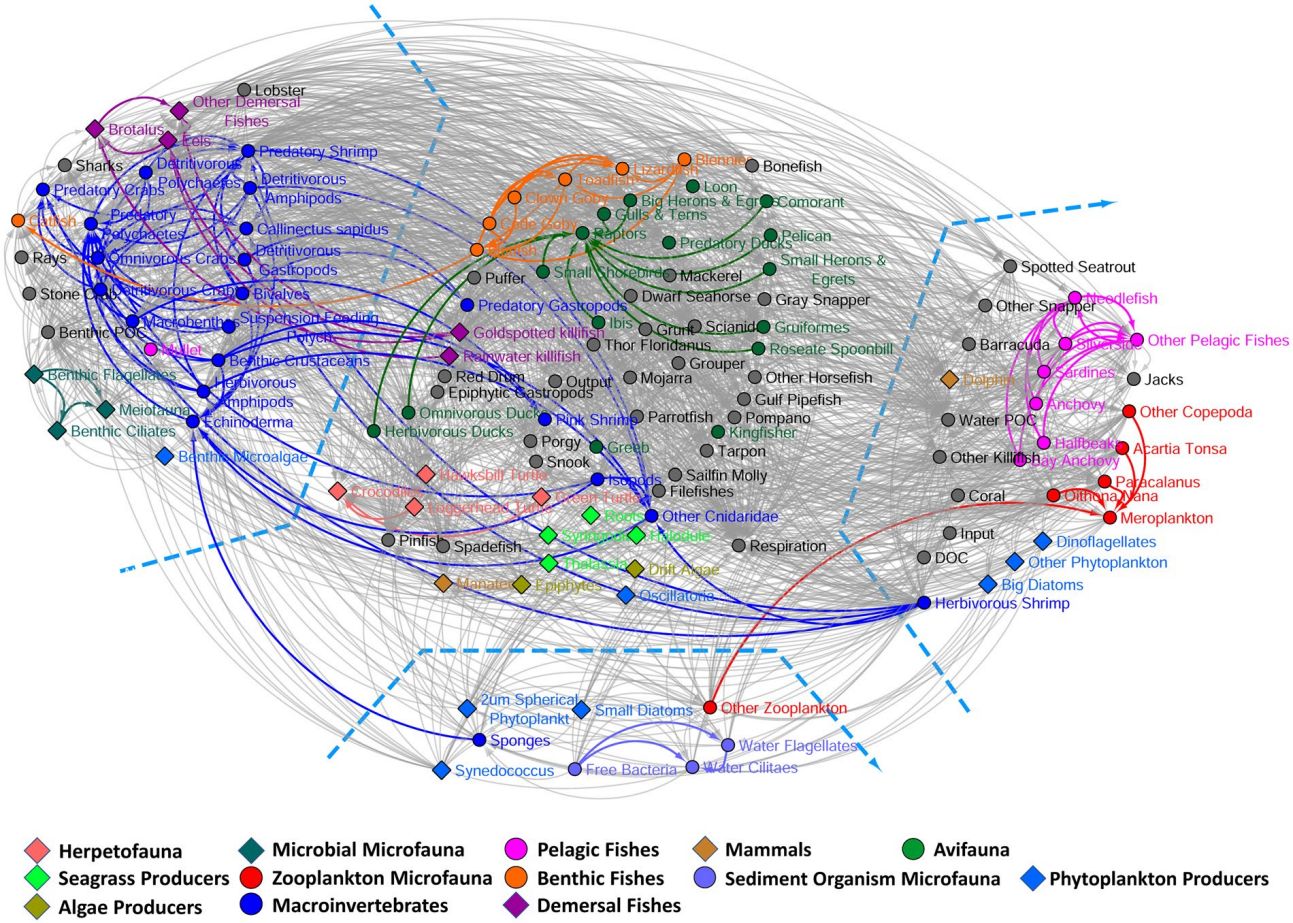
Food web in the Florida Bay. The nodes are colored based on the group classification given in the original research report^60^. The spatial clustering represents communities detected by the proposed method.

Our second example is a food web representing the carbon exchange among 128 compartments (organisms and species) occurring during the wet and dry seasons in the Florida Bay ecosystem^60^, as shown in Fig. 6. In this network, nodes represent compartments, and links represent energy flow (the link from node *i* to node *j* means that carbon is transferred from node *i* to node *j*). Part of the compartments are classified into a total of 13 groups (Part of the groups were compiled by Benson *et al*.^41^), as marked with different colors in Fig. 6. The remaining compartments are left in grey. This network is a directed network, and we apply the proposed method to a simplified version with each directed edge converted to an undirected edge.

The communities detected by the proposed method are divided by the dashed lines. The division corresponds quite closely with the division of groups of compartments. The clustering reveals four known aquatic layers: macroinvertebrates and microbial microfauna (left), sediment organism microfauna (bottom), pelagic fishes and zooplankton microfauna (right), and algae producers, avifauna, benthic fishes, herpetofauna, and seagrass producers (middle). Interestingly, some groups are evenly distributed in multiple communities, like mammals, demersal fishes, and phytoplankton producers, while some other groups have a few members clustered into different communities, like benthic fishes, macroinvertebrates, and pelagic fishes. This phenomenon presumably indicates that the roles of these species in the carbon exchange cannot be derived from the traditional divisions in a trivial manner. For example, though both are mammals, the manatee and the dolphin have very diverse diets. The manatee feeds on submergent aquatic vegetation, and the dolphin feeds on small fishes and shrimps. Consequently, one would expect that the manatee and the dolphin play different roles in the carbon exchange. Thus the simple traditional divisions of taxa, for example, into benthic, demersal, and pelagic organisms, or into fishes, aves, herptiles, and mammals, may not ideally reflect their roles in the carbon exchange.

### Neural Network

**Figure 7:**
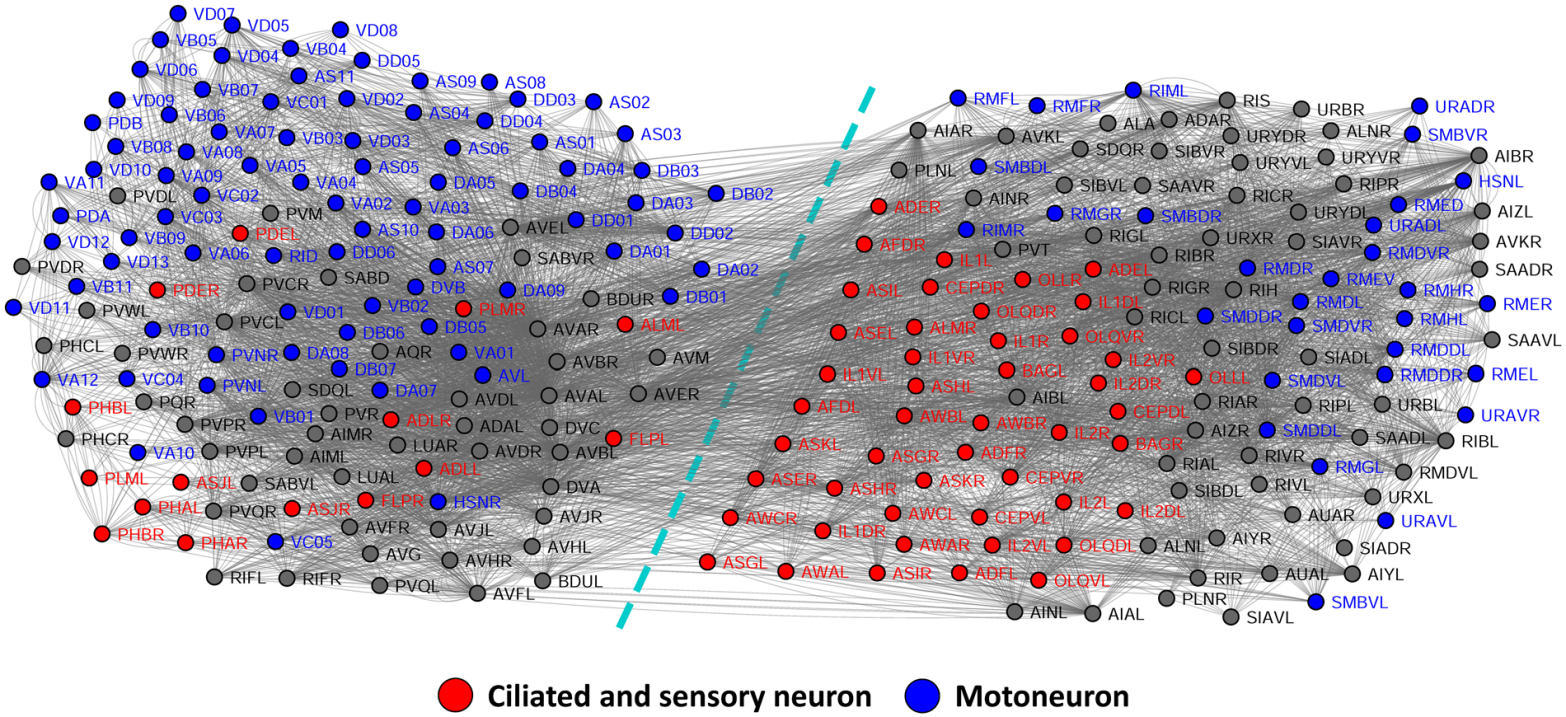
Neural network of the nematode *Caenorhabditis elegans*. The nodes are colored based on neuron categories described in the original research report^61^. The spatial clustering represents communities detected by the proposed method.

Our third example is the nervous system of the soil nematode *Caenorhabditis elegans*^61^, the only organism whose connectome has been completely mapped so far. The nervous system of *C. elegans* is represented by a neural network consisting of 280 nonpharyngeal neurons and covering 6393 chemical synapses, 890 electrical junctions, and 1410 neuromuscular junctions^62,63^, as shown in Fig. 7. In this network, nodes represent neurons and links represent the existence of any of the three neural interactions. The original network is directed and contains multi-edges and loops, and we apply the proposed method to the simplified undirected version, with each directed edge converted to an undirected edge, multi-edges merged, and loops deleted. We have labeled part of the neurons as ciliated/sensory neuron or motoneuron based on descriptions in the original research^61^, and these labeled neurons are colored in Fig. 7. The remaining neurons are left in grey. In general, ciliated/sensory neurons are neurons that are part of sensilla (groups of sense organs) or directly associated with sensilla, and motoneurons are neurons that innervate muscles. The neurons left in grey are mostly interneurons that create neural circuits among other neurons.

The dashed line denotes the community division found by the proposed method. As can be seen, the achieved division yields an approximate distinction between ciliated/sensory neurons and motoneurons. This distinction is not perfect: A small number of ciliated/sensory neurons find their way into the motoneuron community (left), and several motoneurons are clustered into the ciliated/sensory-neuron community (right). This “incorrect” clustering of motoneurons is understandable. The families of motoneurons clustered into the ciliated/sensory-neuron community (RIM, RMD, RME, RMF, RMG, RMH, SMB, SMD, URA) are motoneurons that innervate head muscles and are located near the head, where the major sensilla are also located. Thus one would expect these motoneurons to frequently interact with ciliated/sensory neurons that are also located in the head. On the other hand, part of the families of ciliated/sensory neurons clustered into the motoneuron community (PHB, PHA, PDE, PLM) are ciliated/sensory neurons that are connected to sensilla located at the posterior body, where motoneurons are densely located to control body movements. As a result, one would expect these ciliated/sensory neurons to be more associated with local motoneurons than with ciliated/sensory neurons in the head. However, the other four families of incorrectly clustered ciliated/sensory neurons (ADL, ASJ, ALM, FLP) cannot be explained by this theory, because they are located near the head, and in addition some of them are connected to major sensilla in the head. This anomaly might arise because our simplification of the neural network (ignoring interaction directions, merging multi-edges, deleting loops, and regarding all kinds of neural interactions as equivalent) could only approximately represent neural associations, and some information is lost after the simplification.

## Conclusion and Discussion

In this paper, we developed a novel community-detection method on the basis of cliques, i.e., local complete subnetworks. The proposed method overcomes the deficiencies of previous similar community-detection methods by considering the nested nature of cliques and encoding the size of cliques into the optimization objective function. In addition, it does not require any pre-specification of the type or size of the subnetworks considered in partitioning. To verify the effectiveness of the proposed method, numerical experiments were conducted using both well-established benchmarks and real-world networks with known communities. In all cases, the community structure detected by the proposed method either achieves state-of-the-art performance or aligns well with ground-truth communities. Finally, we applied the clique-based community-detection method to real-world networks with no a priori information regarding community structure. Specifically, the detected community structure provides insights into the social groupings of bottlenose dolphins, the roles of compartments in ecological carbon exchange, and the functions of neurons in the connectome of the model organism *Caenorhabditis elegans*. We also presented a theoretical analysis of the performance of the proposed method. Specifically, we showed that our method was guaranteed to yield near-optimal performance in the bipartition case, and analyzed the computational complexity of our method.

The proposed method emphasizes the power of maximal cliques in community detection. In networks with community structure, nodes within each community tend to be densely interconnected and may potentially form multiple cliques with large sizes, whereas nodes from different communities are sparsely connected and so are unlikely to form high-order cliques. It would in general be unfair to assume that the sizes of these cliques are above some certain threshold, though most existing methods involving cliques have made such assumptions. Maximal cliques allow algorithms to operate without such assumptions by adaptively encoding all clique information based on whatever clique sizes are available. Though the computational complexity of the proposed method makes it unsuitable for large-scale networks, considering maximal cliques could be useful in devising more computationally efficient methods. For example, some greedy methods may converge faster without losing much accuracy by treating local maximal cliques as a whole. By requiring only information of local maximal cliques, it is possible to bypass the collection of global maximal-clique information, which is computationally expensive.

## Theoretical Analysis

In this section, we present the theoretical analysis of the proposed method. We begin by analyzing the performance of the proposed method for a special case. We then discuss the computational complexity of the proposed method, and conclude this section by proving the key theoretical results in this paper.

### Performance Guarantee for Graph Bipartition

For the case *m* = 2, the graph-partitioning problem becomes a graph-bipartition problem. For this special case, spectral graph theory provides guidance on measuring the goodness of approximation to the clique conductance minimization^64–66^. One way is through an expanded version of the Cheeger inequality that characterizes the performance of spectral graph partitioning^67^. We follow a similar approach in the remainder of this subsection. Next we introduce terminology necessary to present our result. Let ${\mathcal{G}}=({\mathcal{V}},{\mathcal{E}},\pi )$ be a connected undirected binary graph with no loops. For a subset $A\subset {\mathcal{V}}$, the Cheeger ratio of *A* is defined as

16 $$h(A)\,:=\,\frac{{\rm{c}}{\rm{u}}{\rm{t}}(A,\bar{A})}{min({\rm{v}}{\rm{o}}{\rm{l}}(A),{\rm{v}}{\rm{o}}{\rm{l}}(\bar{A}))},$$

and the Cheeger constant of ${\mathcal{G}}$ is defined as

17 $${h}_{{\mathcal{G}}}\,:=\,\mathop{min}\limits_{A}\,h(A).$$

Let ${\alpha }_{{\mathcal{G}}}$ be the Cheeger ratio of the output of Algorithm 1. Chung proved an expanded version of the Cheeger inequality, relating these values for spectral bipartition on connected binary graphs^67^. However, in our setting, ${{\mathcal{G}}}_{c}$ is defined to be a weighted graph. Thus our first step is to generalize Chung’s result to connected weighted graphs.

#### Lemma 3.

(Expanded Cheeger inequality). *Let* ${\mathcal{G}}$ *be a connected undirected binary graph and* ${{\mathcal{G}}}_{c}$ *be the induced clique graph with a normalized Laplacian matrix* ${ {\mathcal L} }_{c}$*. Let* ${\lambda }_{{\mathcal{G}}}$ *be the second smallest eigenvalue of* ${ {\mathcal L} }_{c}$*, and* ${h}_{{\mathcal{G}}}$ *be the Cheeger constant of* ${\mathcal{G}}$*. Then*

18 $$2{h}_{{\mathcal{G}}}\ge {\lambda }_{{\mathcal{G}}}\ge \frac{{\alpha }_{{\mathcal{G}}}^{2}}{2}\ge \frac{{h}_{{\mathcal{G}}}^{2}}{2},$$

*where* ${\alpha }_{{\mathcal{G}}}$ *is the Cheeger ratio of the output of Algorithm* 1.

The proof of Lemma 3 is given later in this section. In our setting, the Cheeger constant ${h}_{{\mathcal{G}}}$ is equal to ${{\varphi }}^{* }$, which is the optimal value of the clique conductance optimization (12), and the Cheeger ratio ${\alpha }_{{\mathcal{G}}}$ is equal to $\hat{{\varphi }}$, which is the clique conductance of the output of Algorithm 1. Therefore, combining Proposition 1 and Lemma 3 yields Theorem 4.

#### Theorem 4.

*Let* ${\mathcal{G}}$ *be a connected undirected binary graph. Let* ${{\varphi }}^{* }$ *denote the optimal clique-conductance value of* (12) *and* $\hat{{\varphi }}$ *be the clique-conductance value of output of Algorithm 1 for the case m =* 2. *Then*

19 $${{\varphi }}^{* }\le \hat{{\varphi }}\le 2\sqrt{{{\varphi }}^{* }}\mathrm{.}$$

Theorem 4 shows that our optimization algorithm finds a bipartition that is bounded within the optimal bipartition by a quadratic factor. Therefore our algorithm is mathematically guaranteed to achieve a near-optimal partition.

### Performance Guarantee Verification

To verify the performance guarantee of the proposed method, given in Theorem 4, we apply it to a set of randomly generated graphs. Each graph is composed of *n* vertices, each of which is assigned a random point in [0,1]^100^. An undirected weighted graph is generated by computing the negative Euclidean distances between each pair of these vertices, and then an undirected binary graph is generated by preserving a percentage *ρ* of the edges with the largest weights. This process produces graphs that reflect the degradation of correlation with distance, which is a common assumption in many network models, and that are essentially random in other aspects.

**Figure 8:**
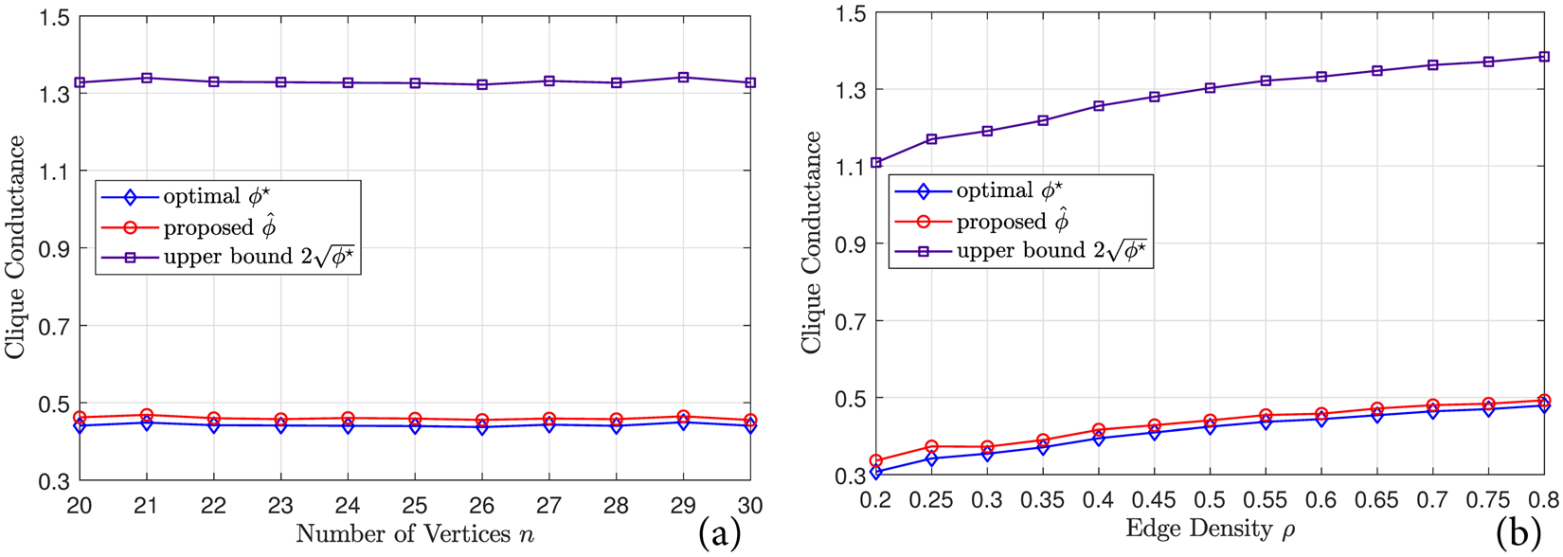
Comparisons of clique conductance of the proposed method and the performance bounds in Theorem 4. (**a**) Varying *n* and fixed *ρ* = 0.6. (**b**) Varying *ρ* and fixed *n* = 30.

We apply the proposed method to each graph and partition it into two parts. We also enumerate all possible bipartitions and find the bipartition with the minimal clique conductance. In Fig. 8a, we show comparisons of clique conductance of the bipartitions achieved by the proposed method and the optimal bipartitions, with *n* varying from 20 to 30 and *ρ* = 0.6. In Fig. 8b, we repeat the experiments with *n* = 30 and *ρ* varying from 0.2 to 0.8. Each curve is averaged over 50 independent trials. As can be seen, the proposed method follows the optimal performance curve closely in general, and is well bounded by the upper bound in Theorem 4. In other words, the proposed method performs almost perfectly and always finds a near-optimal bipartition.

### Computational Complexity

Finding all maximal cliques in an arbitrary graph requires O(3^*n*/3^) computations^45^, which is optimal as a function of *n* because any *n*-vertex graph has up to 3^*n*/3^ maximal cliques^68^. After forming the clique weight matrix, computing the first *m* eigenvectors requires an eigenvalue decomposition of the clique weight matrix, for which the computational complexity is O(*n*^3^)^69^. The *k*-means clustering algorithm needs O(*nm*^2^*i*) computations^70^, where *i* is the number of iterations needed to achieve convergence. Since *m* is much less than *n* and *i* is very small in practice, we conclude that the number of required computations in the clustering scales as O(*n*^3^).

In Table 1, we summarize the computational complexity of the proposed method, the motif-conductance method, and other community-detection methods discussed in the Empirical Results section. As can be seen, the greedy methods are much faster than the proposed method, but the proposed method exhibits better performance on benchmarks (see Fig. 1). The motif-conductance method suffers from the high computational complexity of the brute-force search for the optimal clique size before clustering. By focusing on maximal cliques, the proposed method decreases the computational complexity of this step from O(2^*n*^) to O(3^*n*/3^). However, the exponential complexity of the proposed method still makes it unsuitable for large networks.

**Table 1:** Computational complexity of the community-detection methods.

	Initialization	Clustering
Proposed	O(3^*n*/3^)	O(*n*^3^)
Motif^41^	O(2^*n*^)	O(*n*^3^)
Spectral^34^	—	O(*n*^3^)
Louvain^48^	—	O(*nlogn*)
Ravasz^49^	—	O(*n*^2^)
Fast Modularity^50–52^	—	O(*n*(*logn*)^2^)

**Proof of Proposition 1**

#### *Proof*.

Let ***z*** ∈ {0, 1}^*n*^ be a vector such that ***z***(*i*) = 1 if *v*_*i*_ ∈ *A* and ***z***(*i*) = 0 if ${v}_{i}\in \bar{A}$. Further let **W**_*c*,*k*_ be an adjacency matrix defined as

$${{\bf{W}}}_{c,k}(i,j)\,:\,=\,\sum _{\sigma \in { {\mathcal M} }_{k}}\sum _{{v}_{i},{v}_{j}\,\in \,\sigma }\omega (\sigma ),$$

let **D**_*c*,*k*_ be the corresponding degree matrix, and let **L**_*c*,*k*_ be the corresponding Laplacian matrix. Then

$$\begin{array}{ccc}{\rm{c}}{\rm{u}}{\rm{t}}(A,\bar{A}) & = & \sum _{k\ge 1}\,\sum _{\sigma \in {{\mathcal{M}}}_{k}}\omega (\sigma )\sum _{{v}_{i},{v}_{j}\in \sigma }{\mathbbm{1}}(z(i)=1,z(j)=0)\\  & = & \frac{1}{2}\sum _{k\ge 1}\,\sum _{\sigma \in {{\mathcal{M}}}_{k}}\omega (\sigma )\sum _{\{{v}_{i},{v}_{j}\}\subset \sigma }{\mathbbm{1}}(z(i)\ne z(j))\\  & = & \frac{1}{2}\sum _{k\ge 1}\,\sum _{\sigma \in {{\mathcal{M}}}_{k}}\omega (\sigma )\sum _{\{{v}_{i},{v}_{j}\}\subset \sigma }{(z(i)-z(j))}^{2}\\  & = & \sum _{k\ge 1}{z}^{{\rm{T}}}{{\bf{L}}}_{c,k}z\\  & = & {z}^{{\rm{T}}}{{\bf{L}}}_{c}z\\  & = & {{\rm{c}}{\rm{u}}{\rm{t}}}_{c}(A,\bar{A}),\end{array}$$

where the fourth and sixth equalities make use of the standard properties of Laplacian matrices^30^, and the fifth equality follows ${{\bf{L}}}_{c}={\sum }_{k}{{\bf{L}}}_{c,k}$. In addition,

$$\begin{array}{ccc}{\rm{v}}{\rm{o}}{\rm{l}}(A) & = & \sum _{k\ge 1}\,\sum _{\sigma \in {{\mathcal{M}}}_{k}}\omega (\sigma )\sum _{{v}_{i}\in \sigma }z(i)\\  & = & \sum _{k\ge 1}{z}^{{\rm{T}}}{{\bf{D}}}_{c,k}z\\  & = & {z}^{{\rm{T}}}{{\bf{D}}}_{c,k}z\\  & = & {{\rm{v}}{\rm{o}}{\rm{l}}}_{c}(A),\end{array}$$

where the third equality follows from ${{\bf{D}}}_{c}={\sum }_{k}\,{{\bf{D}}}_{c,k}$. This concludes the proof.☐

**Proof of Lemma 3**

#### *Proof*.

This proof extends Chung’s proof to connected weighted graphs^67^. The second smallest eigenvalue ${\lambda }_{{\mathcal{G}}}$ of ${ {\mathcal L} }_{c}$ can be expressed as the infimum of the Rayleigh quotient

20 $${\lambda }_{{\mathcal{G}}}=\mathop{inf}\limits_{{\boldsymbol{y}}}\,R({\boldsymbol{y}})=\mathop{inf}\limits_{{\boldsymbol{y}}}\frac{{\sum }_{u\sim v}{({\boldsymbol{y}}(u)-{\boldsymbol{y}}(v))}^{2}{\pi }_{c}(u,v)}{{\sum }_{v\in {\mathcal{V}}}{\boldsymbol{y}}{(v)}^{2}{d}_{v}},$$

where $u\sim v$ means {*u*, *v*} is a connected pair of vertices, and ***y*** satisfies ${\sum }_{v\in {\mathcal{V}}}{\boldsymbol{y}}(v){d}_{v}=0$. Suppose the Cheeger constant, ${h}_{{\mathcal{G}}}$, is achieved by a set *S*. Let *χ*_*S*_ be the vectorized indicator function of *S*, defined as

$${\chi }_{S}(u)=\{\begin{array}{cc}1 & {\rm{i}}{\rm{f}}\,u\in S,\\ 0 & {\rm{o}}{\rm{t}}{\rm{h}}{\rm{e}}{\rm{r}}{\rm{w}}{\rm{i}}{\rm{s}}{\rm{e}}.\end{array}$$

Consider ${\boldsymbol{y}}={{\chi }}_{S}-{\rm{v}}{\rm{o}}{\rm{l}}(S)/{\rm{v}}{\rm{o}}{\rm{l}}({\mathcal{V}}){\bf{1}}$, and it follows that

21 $${\lambda }_{{\mathcal{G}}}\le R({\boldsymbol{y}})\le 2{h}_{{\mathcal{G}}}.$$

Thus the remainder of this proof focuses on deriving a lower bound for ${\lambda }_{{\mathcal{G}}}$ in terms of Cheeger ratios.

Let ***g*** be an eigenvector achieving ${\lambda }_{{\mathcal{G}}}$, namely,

22 $${\boldsymbol{g}}=\mathop{{\rm{\arg }}\,{\rm{\min }}}\limits_{{{\boldsymbol{y}}}^{{\rm{T}}}{{\bf{D}}}_{c}{\bf{1}}=0}\,\frac{{{\boldsymbol{y}}}^{{\rm{T}}}({{\bf{D}}}_{c}-{{\bf{W}}}_{c}){\boldsymbol{y}}}{{{\boldsymbol{y}}}^{{\rm{T}}}{{\bf{D}}}_{c}{\boldsymbol{y}}}\mathrm{.}$$

Reorder the vertices such that

$${\boldsymbol{g}}({v}_{1})\ge {\boldsymbol{g}}({v}_{2})\ge \cdots \ge {\boldsymbol{g}}({v}_{n}),$$

and set *S*_*i*_ = {*v*_1_, …, *v*_*i*_}. It follows that

23 $${\alpha }_{{\mathcal{G}}}=\mathop{min}\limits_{i}\,h({S}_{i}).$$

Let *r* denote the largest integer such that ${\rm{v}}{\rm{o}}{\rm{l}}({S}_{r})\le {\rm{v}}{\rm{o}}{\rm{l}}({\mathcal{V}})/2$. Since ***g***^T^**D**_*c*_**1** = 0,

$$\sum _{i=1}^{n}{\boldsymbol{g}}{(i)}^{2}d=\mathop{min}\limits_{c}\sum _{i=1}^{n}{({\boldsymbol{g}}(i)-c)}^{2}{d}_{i}\le \sum _{i=1}^{n}{({\boldsymbol{g}}(i)-{\boldsymbol{g}}({s}_{r}))}^{2}{d}_{i},$$

where *d*_*i*_ := **D**_*c*_(*i*, *i*) for any *i*. Denote by ***g***_+_ and ***g***_−_ the positive and negative parts of ***g*** − ***g***(*s*_*r*_), respectively, defined as

$$\begin{array}{ccc}{{\boldsymbol{g}}}_{+}(i) & = & \{\begin{array}{cc}{\boldsymbol{g}}(i)-{\boldsymbol{g}}({s}_{r}) & {\rm{i}}{\rm{f}}\,{\boldsymbol{g}}(i)\ge {\boldsymbol{g}}({s}_{r}),\\ 0 & {\rm{o}}{\rm{t}}{\rm{h}}{\rm{e}}{\rm{r}}{\rm{w}}{\rm{i}}{\rm{s}}{\rm{e}},\end{array}\\ {{\boldsymbol{g}}}_{-}(i) & = & \{\begin{array}{cc}{\boldsymbol{g}}({s}_{r})-{\boldsymbol{g}}(i) & {\rm{i}}{\rm{f}}\,{\boldsymbol{g}}(i)\le {\boldsymbol{g}}({s}_{r}),\\ 0 & {\rm{o}}{\rm{t}}{\rm{h}}{\rm{e}}{\rm{r}}{\rm{w}}{\rm{i}}{\rm{s}}{\rm{e}}.\end{array}\end{array}$$

By the Rayleigh-Ritz theorem^71^,

$$\begin{array}{ccc}{\lambda }_{{\mathcal{G}}} & = & R({\boldsymbol{g}})\\  & = & \frac{{\sum }_{u\sim v}{({\boldsymbol{g}}(u)-{\boldsymbol{g}}(v))}^{2}{\pi }_{c}(u,v)}{{\sum }_{v\in {\mathcal{V}}}{\boldsymbol{g}}{(v)}^{2}{d}_{v}}\\  & \ge  & \frac{{\sum }_{u\sim v}{({\boldsymbol{g}}(u)-{\boldsymbol{g}}(v))}^{2}{\pi }_{c}(u,v)}{{\sum }_{v\in {\mathcal{V}}}{({\boldsymbol{g}}(v)-{\boldsymbol{g}}({v}_{r}))}^{2}{d}_{v}}\\  & \ge  & \frac{{\sum }_{u\sim v}({({{\boldsymbol{g}}}_{+}(u)-{{\boldsymbol{g}}}_{+}(v))}^{2}+{({{\boldsymbol{g}}}_{-}(u)-{{\boldsymbol{g}}}_{-}(v))}^{2}){\pi }_{c}(u,v)}{{\sum }_{v\in {\mathcal{V}}}({{\boldsymbol{g}}}_{+}{(v)}^{2}+{{\boldsymbol{g}}}_{-}{(v)}^{2}){d}_{v}}.\end{array}$$

Without loss of generality, we may assume *R*(***g***_+_) ≤ *R*(***g***_−_), and then we have ${\lambda }_{{\mathcal{G}}}\ge R({{\boldsymbol{g}}}_{+})$ because

$$\frac{a+b}{c+d}\ge \,min(\frac{a}{c},\frac{b}{d})$$

if *a*, *b*, *c*, *d* > 0. For ease of presentation, we use the notation ${{\rm{vol}}}^{\dagger }(S)\,:\,=\,{\rm{\min }}({\rm{vol}}(S),{\rm{vol}}(\bar{S}))$. Then we have

$$\begin{array}{ccc}{\lambda }_{{\mathcal{G}}} & \ge  & R({{\boldsymbol{g}}}_{+})\\  & = & \frac{{\sum }_{u\sim v}{({{\boldsymbol{g}}}_{+}(u)-{{\boldsymbol{g}}}_{+}(v))}^{2}{\pi }_{c}(u,v)}{{\sum }_{v\in {\mathcal{V}}}{{\boldsymbol{g}}}_{+}{(v)}^{2}{d}_{v}}\\  & = & \frac{({\sum }_{u\sim v}{({{\boldsymbol{g}}}_{+}(u)-{{\boldsymbol{g}}}_{+}(v))}^{2}{\pi }_{c}(u,v))\,({\sum }_{u\sim v}{({{\boldsymbol{g}}}_{+}(u)+{{\boldsymbol{g}}}_{+}(v))}^{2}{\pi }_{c}(u,v))}{{\sum }_{v\in {\mathcal{V}}}{{\boldsymbol{g}}}_{+}{(v)}^{2}{d}_{v}{\sum }_{u\sim v}{({{\boldsymbol{g}}}_{+}(u)+{{\boldsymbol{g}}}_{+}(v))}^{2}{\pi }_{c}(u,v)}\\  & \ge  & \frac{{({\sum }_{u\sim v}({{\boldsymbol{g}}}_{+}{(u)}^{2}-{{\boldsymbol{g}}}_{+}{(v)}^{2}){\pi }_{c}(u,v))}^{2}}{2{({\sum }_{v\in {\mathcal{V}}}{{\boldsymbol{g}}}_{+}{(v)}^{2}{d}_{v})}^{2}}\\  & = & \frac{{({\sum }_{1\le i\le n-1}|{{\boldsymbol{g}}}_{+}{({v}_{i})}^{2}-{{\boldsymbol{g}}}_{+}{({v}_{i+1})}^{2}|{\rm{c}}{\rm{u}}{\rm{t}}({S}_{i},{\bar{S}}_{i}))}^{2}}{2{({\sum }_{v\in {\mathcal{V}}}{{\boldsymbol{g}}}_{+}{(v)}^{2}{d}_{v})}^{2}}\\  & \ge  & \frac{{({\sum }_{1\le i\le n-1}|{{\boldsymbol{g}}}_{+}{({v}_{i})}^{2}-{{\boldsymbol{g}}}_{+}{({v}_{i+1})}^{2}|{a}_{{\mathcal{G}}}{{\rm{v}}{\rm{o}}{\rm{l}}}^{\dagger }({{\boldsymbol{S}}}_{i}))}^{2}}{2{({\sum }_{v\in {\mathcal{V}}}{{\boldsymbol{g}}}_{+}{(v)}^{2}{d}_{v})}^{2}}\\  & = & \frac{{\alpha }_{{\mathcal{G}}}^{2}}{2}\frac{{({\sum }_{1\le i\le n}{{\boldsymbol{g}}}_{+}{({v}_{i})}^{2}|{{\rm{v}}{\rm{o}}{\rm{l}}}^{\dagger }({S}_{i})-{{\rm{v}}{\rm{o}}{\rm{l}}}^{\dagger }({S}_{i-1})|)}^{2}}{({\sum }_{v\in {\mathcal{V}}}{{\boldsymbol{g}}}_{+}{(v)}^{2}{d}_{v}{)}^{2}}\\  & = & \frac{{\alpha }_{{\mathcal{G}}}^{2}}{2}\frac{{({\sum }_{1\le i\le n}{{\boldsymbol{g}}}_{+}{({v}_{i})}^{2}{d}_{{v}_{i}})}^{2}}{{({\sum }_{v\in {\mathcal{V}}}{{\boldsymbol{g}}}_{+}{(v)}^{2}{d}_{v})}^{2}}\\  & = & \frac{{\alpha }_{{\mathcal{G}}}^{2}}{2}\end{array}$$

where the second inequality is by the Cauchy-Schwarz inequality and the arithmetic-geometric-mean inequality, and the third inequality is by definition of ${\alpha }_{{\mathcal{G}}}$. This concludes the proof.☐
