# Publisher Correction: Community Detection in Complex Networks via Clique Conductance

**DOI:** 10.1038/s41598-018-29006-4

**Published:** 2018-07-19

**Authors:** Zhenqi Lu, Johan Wahlström, Arye Nehorai

**Affiliations:** 1 Preston M. Green Department of Electrical and Systems Engineering, Washington University in St. Louis, St. Louis, MO USA; 2 Department of Computer Science, University of Oxford, Oxford, United Kingdom

Correction to: *Scientific Reports* 10.1038/s41598-018-23932-z, published online 13 April 2018

In Algorithm 1, line 12, the superscripted number 48 should not be present. The correct Algorithm 1 appears below.

**Algorithm 1:**
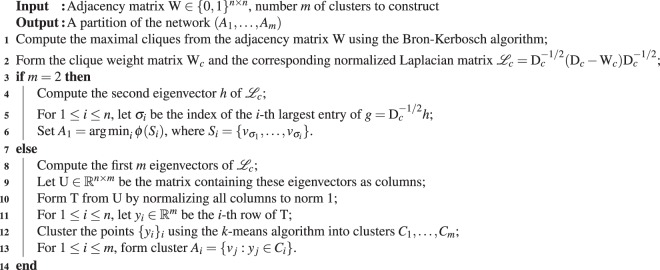
graph partitioning via clique conductance minimization

